# Trusting the Dentist—Expecting a Leap of Faith vs. a Well-Defined Strategy for Anxious Patients

**DOI:** 10.3390/dj10040066

**Published:** 2022-04-07

**Authors:** Rod Moore

**Affiliations:** Institute of Dentistry and Oral Health, Aarhus University, 8000 Aarhus, Denmark; rod.moore@dent.au.dk

**Keywords:** trust, dentist-patient relationship, dental anxiety, person-centred dentistry, patient-centred dentistry

## Abstract

This article aimed to set into perspective the unique aspects of trust within the dentist–patient relationship by exploring the literature as well as historical aspects of dentistry in the association between trust/distrust and patient anxiety. In order to characterise this uniqueness, the assumptions for trusting in dentistry are compared and contrasted with other professions using a conceptual analysis. The professions of medicine, sociology, psychology, nursing and dentistry were check listed according to the tenets of a concept analytical approach reported by Hupcey et al., in 2001. Recommendations for patient/person-centred care, as opposed to dentist-centred care, that would improve trust are specified according to the literature. These include empowering patients, practicing active listening, empathy and relationship building that might benefit dental patients in relation to the perceived risks of anxiety or induced pain. It was concluded that global distrust of dominating dentists must give way to person-centred professional strategies so that dentists and patients can tackle their dental anxiety-trust challenges, both in the public’s image of the dental profession and in clinical relationships. Future directions would be to explore incentives for dentists to change to patient/person-centred care.

## 1. Introduction

The concept of trust seems naturally woven into the fabric of the dentist–patient relationship. Trust or distrust in the relationship appears to correlate highly with patient pain and anxiety perceptions while also being sources of negative stress for dentists [1,2]. In spite of evidence of these relationships, which are multifaceted and complex, associations of trust/distrust with other factors and variables over time are still poorly understood as is their causal relationship with pain and anxiety perceptions [3].

A 2017 review of the literature pertaining to trust and the dental profession reveals a paucity of discussion with respect to the tenets of trust and is almost exclusively focused on clinical outcomes related to any dentist–patient relationship [3]. These can also be strongly affected by other associated conceptual variables, such as satisfaction with or economics of the health care system [3,4,5,6,7]. Only recently has the use of a psychometric measure of trust in dentists been validated and employed [3,8]. Armfield et al. [3] showed that low trust of dentists was significantly associated with adverse outcomes, including current dental pain often associated with increased dental avoidance, and higher dental anxiety. Armfield et al. [3] surmised that whether lower trust played a specific directional causal role in these associations was not yet determinable given their cross-sectional study. However, given the assumptions of this unidimensional 11-item Dentist Trust Scale, at least they indicated a conceptual causal pathway between low levels of trust and dental anxiety. They pointed out that people with relatively less trust in dentists generally were found to be significantly more likely to have experienced discomfort in previous treatment, feelings of gagging, faintness, light-headedness, embarrassment or having personal problems with the dentist during treatment. More recently, Wu et al. [9,10] also found in a Chinese population that persons who had never attended the dentist had negative perceptions of dentists, indicating problems with the public image of dental professionals. Irregular and never-attenders were also most often to have high dental anxiety [9,10]. Several researchers [2,11,12,13] had earlier presented data and argued that negative dentist and staff behaviour and distrust are important factors in provoking anxiety. Furthermore, establishing rapport and trust were seen as key elements in creating a positive patient–dentist relationship. Milgrom et al. [12] and Moore et al. [2] argued that the foundation of psychological management of dental anxiety is namely for the dentist to build such a trusting relationship with the patient.

In a British study with over 11,000 subjects published in 2020, Yuan et al. [14] supported these findings when they found that dental anxiety was predicted negatively by person’s trust and shame and positively by reported dentist communication. Thus, this larger British study verified a logical hypothesis that negative past experiences were associated with dental anxiety, especially those specifically concerning practitioner behaviour, which resulted in reduced trust. This implies that interpersonal communication is more important in establishing trust in the dentist than in other experiences associated with the treatment [2,3,14] Armfield et al. [3] had also shown that there was no significant association between trust scores and having previously experienced pain. They suggested [2,3] that providing a pain-free experience, while important, may be less important than how the dentist responds when a patient does experience pain.

Armfield et al. [3] and Yuan et al. [14] thus attempted, in these two recent studies, to conceptualise trust in dentist–patient relationships and its correlates with dental anxiety. Communication style and satisfactory outcomes were their main conceptual foci. However, neither of these studies had the aim of comparing or contrasting the state of the science of discipline-specific conceptualizations of trust and the uniqueness of trust/distrust outcomes compared with other professions. Such comparisons or contrasts would support the uniqueness of conditions for trust in dentist–patient relationships, especially since it appears to be associated with dental anxiety.

Hupcey et al. [6] in 2001 conducted trust concept comparisons and contrasts across disciplines with a conceptual analysis of the contemporary literature that included medicine, psychology, sociology and her own profession—nursing. Hupcey et al. [6] posited that interdisciplinary conceptualization advances a common concept toward maturity—that is, a more refined, pragmatic and higher-order concept. The Hupcey et al. [6] concept study stands alone 20 years later [15] as the only example of an analytical approach to identify conceptual components of trust. The aim of the present paper was to revisit the reasoning of Hupcey et al. [6] and to apply it to the concept of trust in dentist–patient relationships, given the uniqueness of the profession’s literature and historical development.

## 2. Methods

### 2.1. Derivation of the Conceptual Components of Trust Re. Patient/Dentist/Anxiety

According to Hupcey et al. [6], the conceptual components of trust are antecedents or preconditions, attributes, boundaries and outcomes. Each are identifiable and autonomous. Although there are notable differences among the four disciplines, they studied the structural features or conceptual components of trust. They discovered theoretical commonalties that transcended all four disciplines. From this interdisciplinary perspective, they developed a theoretical definition of trust [6]. These conceptual components are described below using patient/dentist/anxiety examples to illustrate them.

#### 2.1.1. Antecedents of Trust

Hupcey et al. [6] first described antecedents or preconditions to trust, which require the following: (1) a need that cannot be met without the help of another; (2) prior knowledge and/or experience with the other/profession; and (3) some assessment of risk or what is at stake. An example scenario includes the following: A cavity in a patient’s tooth requires special instruments and procedures to fix it that the patient does not master. Dentists are known for providing these forms of technical assistance. If a patient is afraid of dentists based on previous bad experiences with one particular dentist, the patient must try to assess the risk that another dentist, in other circumstances, might reinforce their fear. Armfield et al. [3] determined that the global trust of dentists is difficult to separate from trust of specific dentists and that this is inversely correlated with dental anxiety. Thus, when a person has dental anxiety, this seems to be an antecedent to distrust of dentists due to conditioning from bad experiences, which is most often the case [2,3,14].

#### 2.1.2. Attributes of Trust

As alluded to above, trust involves a person’s willingness to place him or herself in a situation of risk: that is, a vulnerable situation in which the outcome may not be what is expected. There is also an expectation that the trusted person/professional will behave in a manner that helps meet the identified need. Finally, testing the trustworthiness of the person in whom trust is placed over time is also involved. In summary, Hupcey et al. [6] describe the attributes of trust as follows: (1) dependency on another individual to have a need met; (2) choice or willingness to take some risk; (3) an expectation that the trusted individual will behave in a certain way; (4) limited focus to the area or behaviour related to the need; and (5) testing of the trustworthiness of the individual. An example scenario includes the following: A fearful patient must depend on a dentist not only to help remove a cavity but must also to communicate with the patient in a manner such that the patient will accept the risk that another bad experience with tooth drilling in a similar cavity could be the outcome. However, after having changed dentists three years ago, experiences with the dentist’s treatments and communication skills have all been positive. The patient perceives the new dentist as trustworthy.

#### 2.1.3. Boundaries of Trust

Hupcey et al. [6] also described other concepts that are closely related to trust but do not have all the attributes of trust: faith, confidence and risk-taking. Faith is a concept that is not necessarily based on prior evidence that a person/professional will behave in a certain way, nor does faith involve testing or choice [6]. Confidence does not involve testing or placing oneself in a dependent situation [6]. The concept of risk-taking is similar to trust, except that risk-taking benefits do not always outweigh the risk. In summary, Hupcey et al. [6] described that the boundaries of trust are, therefore, based on when trust ceases to exist, which involves the following: (1) the decision to place oneself in a dependent or vulnerable position is not based on some assessment of risk; (2) there is a perception of no choice; and (3) the risks outweigh the benefits. An example scenario includes the following: A patient has avoided dental treatment for twenty-three years due to anxiety. This phobic avoidance, which was recently discovered by her family, has caused them to be on the lookout for dentists advertising that they are specialists in treatment of dental anxiety, since she does not trust the family’s dentist and never contacted him. She makes an appointment with one of the dentists claiming to be a specialist without checking with other professionals or patients to hear about outcomes from their perspective. She just wants to “get it over with.” In the first appointment, after a sleepless night, the patient meets the dentist who sits her in the chair and proceeds to excavate larger cavities that have been causing pain, hoping to place temporary fillings. Armed with only local anaesthesia and soft music in the background, which he points out, the dentist appears to have no special communication skills or training. The patient leaves the operatory in the middle of an excavation due to operative pain and avoids dental treatment for another five years.

#### 2.1.4. Outcomes of Trust

The outcomes of trusting can be positive or negative depending on whether the truster’s expectations were unmet, met or exceeded [6]. These outcome assessments are based on the behaviour of the trusted in relation to meeting the identified need. Satisfaction differs from trust in that it is essentially retrospective, whereas trust reflects an expectation of the quality of an ongoing relationship [16,17]. If there is a positive outcome, then there is a congruence between the initial expectations of the truster and the behaviours of the trusted, and this is correlated with satisfaction as another separate unidimensional concept [4]. The outcome of trust is an evaluation of the congruence between expectations of the trusted person and their actual behaviours. Based on the description in the example scenario above, the woman seeking a cure for her dental anxiety and dental problems was taking a leap of faith based on family advice. She perceived the risk of a negative outcome in seeking a “specialist” to be less than in seeking the family’s dentist with whom she had had no previous contact. Nonetheless, her second bad experience with a dentist was incongruent with her expectations of how a specialist might help her. Her distrust of the first dentist was reinforced by the second, and her distrust of dentists as a profession was also likely negatively reinforced.

Based on this exploration of trust concept development, Hupcey et al. [6] proposed an interdisciplinary scientific definition of trust based on the literature about trust in medicine, psychology, sociology and nursing.

“*Trust emerges from the identification of a need that cannot be met without the assistance of another and some assessment of the risk involved in relying on the other to meet this need. Trust is a willing dependency on another’s actions, but it is limited to the area of need and is subject to overt and covert testing. The outcome of trust is an evaluation of the congruence between expectations of the trusted person and their actions.*”

The literature discussed above and the following discourse about the historical background of the dentist–patient relationship, dental anxiety and trust were used to explore the tenets of Hupcey et al.’s definition and the analytical process from which it was derived in its application to dentistry.

#### 2.1.5. The Historical Background of Dentist-Patient Trust and Anxiety about Dental Treatment

In the European Middle Ages, no health treatment was more satirised or even ridiculed than dental treatment. It was not more than two hundred years ago that the dentist was a traveling showman or “tooth drawer” who pulled teeth on stage for local towns and villages, of course expecting to be paid for services [18,19,20]. Patients were expected to be reluctant and vulnerable objects in these theatrical exhibitions, who had to pay for the humiliating privilege of relief from toothache pain [2]. Alternatively, the rural blacksmith or urban physician would extract teeth, if necessary, in more everyday surroundings and with less fanfare so as not to detract from their primary business interest. The lay traditions of wandering dental practitioners reached their height in the 17th and 18th centuries. Even though, e.g., the French parliament in 1699 set an example for the Western world by requiring dental practitioners, among others, to be examined by a committee of surgeons before being permitted to practice in Paris, traveling dentists pervaded even well into the 19th century, especially in rural areas [20]. Barbers then initiated a tradition of dental surgery, including an apprentice period for young practitioners. Eventually schools of barbering and dentistry would evolve and apprentices shared both tasks [20]. It was only with the advent of responsible physicians who had come to specialise in dentistry that creditable colleges of dental surgery were founded in the mid-1800s with the first school of dental surgery in Baltimore, Maryland, in 1840 [18,20].

##### Modern Society and the Image of the Dentist

As described earlier, the global trust of dentists in societies is difficult to separate from individual perceptions of a specific dentist–patient relationship [2,3]. In modern society, polls have consistently shown that dentists are highly respected professionals and that most patients are satisfied with their own dentist [21,22,23]. Although it indicates positive outcomes, satisfaction with one’s own dentist is inadequate in itself to describe trust in dentists [4]. So how much has the profession’s earlier mercantile image as roving “tooth drawers described above changed over time? Such extreme folklore appears no longer to be commonplace in modern society. However, given the historical background above, the spectacles of pain of treatment and fear still seem imprinted in the social image of dentistry and are engrained within the collective conscience of the population at large to some degree or another, especially as portrayed in mass media, as we shall see below [19,24,25]. Most dental historians insist that these images have remained largely unchanged, since it is only in the last six decades that tooth extraction has become a less appropriate treatment for most dental ailments and only as a “last resort” [19,24]. It appears then that the public’s relations to the dentist may be governed by implicit beliefs about social roles that affect the way one is expected to act as a dental patient and the ways one is expected to be treated by a dentist [26]. This popular “culture of dentistry”, as first described by sociologist Peter Davis [26], describes routines in which a patient–dentist encounter is filled with impressions of: “the white coat, the passive patient, a lack of verbal communication and an overwhelming impression of orderliness, restraint and formality which all seem to be superfluous, if not directly counter, to the technical requirements of the task at hand.”

Nettleton [27] described a sociological and historical perspective of the changes in the context and circumstance for the dentist–patient relationship. She pointed out that, at the beginning of the twentieth century, the dental profession saw pain in treatment as solely a physical response to procedures that involved an intervention on the human body—a physiological and biomedical fact that was required in order to fix a biomedical problem. Such a problem was often endogenous pain, such as toothache, in which, of course, some operational dental treatment pain was acceptable, considering the significant pain relief obtained. However, in other more preventive procedures such as tooth cleanings and fillings, dentist-inflicted pain was comparably less acceptable [27]. The advent of nitrous oxide and ether sedation had started in the 1840s. By the end of the 19th century, it was widely known that painless treatment was possible, at least for tooth extractions and surgeries [27]. Nevertheless, the use of anaesthesia in general dental practice was not widespread in the 19th century. Only after the First World War did dentistry begin to discover that patients began to respond differently [27,28], due to the increasing use of local anaesthetics. With the discovery of procaine (Novocaine) in 1905 and the cylinder ampule in 1917, pain control had become common in dental practices [18]. By 1935, it was proclaimed in an article in the *British Dental Journal*: “…the old idea of the manipulation in the mouth almost regardless of the feelings of the patient has gone, and rightly so, forever. We are at the dawn of a new era of sympathetic dentistry.” [28]. It was believed that once pain was eradicated, fear of treatment also would subside, assuming that the reason people feared dentistry was that they associated it with pain [29]. This meant that the profession perceived that patients were less willing to bear pain inflicted by dental treatment, were more frightened of being hurt, and were developing a “dental conscience” [28] by which to evaluate their trust in each dentist and the profession as a whole. Best also noted in 1935 that pain was “one of our greatest problems, the mastery of which contributes profoundly to professional success.” [30]. The mind of the patient had come to the attention of the dentist and not just their teeth [28]. A new conceptualization of pain and fear (of inflicted pain) involved not only creation of a subjective, feeling patient but also required a sensitive and caring dentist—a new perspective on trust in the dentist–patient relationship [27].

Use of anaesthetic techniques was often not sufficient, because emotional responses to pain appeared sometimes to be impervious to anaesthetic. Suddenly it became apparent that the way in which dentists administered pain control was also essential. Dentists came to wonder, with local anaesthesia failure, whether the physiological vs. psychological or social components needed the most attention. The usual response was a “shot-gun approach”, with primarily anatomical or physiological anaesthetic solutions emerged as the main domain for solving the pain problem and reinforced a narrow biomedical model of pain. Pain experienced by the dental patient required more than just knowledge and facts about physical complaints about teeth. Pain could be experienced without apparent physical cause [27] in parallel with the changes in which patients were becoming increasingly more sceptical about the authority of the dentist. This new social conceptualization of pain and fear in the 1960s and 1970s not only created patient needs for a new, more sensitive and caring dentist in order to manage anxiety and pain but also brought dentists to either respond by changing their behaviour or by sedating patients [27]. Aware of this dilemma, Epstein [31] argued in 1964 that “Overwhelming the patient with sedatives or hypnotic drugs, which destroy the capacity to resist, is not pain control, nor is administering additional narcotizing drugs to control movement so that dental procedures can be performed… (To ensure) the effectiveness of drugs used in pain control, one cannot discount the importance of the manner by which the drugs are administered.” Dentists, again, had to become aware of the fact that they were “…treating humans and not just teeth” [31]. Patients’ acceptance of possibly painful treatment appeared to depend more on the rapport the dentist established with them than on any measure of technical proficiency [31]. Thus, Nettleton surmised that the concept of pain was caught in a polemic in the 1960s and 1970s [27], the decades of the birth of anti-authoritarianism. Nettleton pointed out this historical dichotomy between patients’ resistance to dentistry and their distrust on the one hand and on the other conscious sedation of patients as a type of dentist resistance to role change [27]. This polemic confirmed that the concept of pain from clinical procedures had moved into a social paradigm.

Lack of control over patients’ personal emotional reactions [32,33] and over the social situation in the dental chair [2,34,35,36,37] came to define the profession’s image in societal portrayals displayed in movies, newspapers and books, as described above. Feelings of powerlessness in the dental chair became symbolic of the relationship [38,39]. Thus, images of travelling tooth extraction spectacles from the Middle Ages may not have died out completely in spite of modern technological advances in pain control. Interactions with dentists can still be difficult and embarrassing for fearful patients, causing low self-confidence and feelings of distrust and, at the same time, a bad conscience and emotional conflicts about being so troublesome, especially since they often have avoided treatment for longer periods of time [2].

To summarise the historical context and arguments above, patients have a primary dependence on the technical skills of the dentist, which requires that they trust the dentist and acquiesce to or cooperate with the dentist’s working conditions. The communication is often one-way. If the patient encounters difficulties in feeling secure with this more active and often dominant dentist role, it requires the dentist to demonstrate responsive managerial skills and, in many cases, nurturing or reassuring behaviour. Often without any conscious effort on the part of dentists, technical and procedural demands reduce the prospect for friendly contact and increase the social distance between them and patients [2,40]. This allows dentists to fully concentrate on what they are trained in and expected to do, especially given the time restraints of modern dental practice. The average patient can cope with often confining features of this very special social relationship without much problem, not questioning the dentist’s more active or dominant role. They can exhibit behaviours that facilitate treatment. In patients with high or phobic dental anxiety, however, this scenario of dentist dominance and patient passivity can seem more exaggerated and extreme in their perceptions. Since feelings of patients are their own, the dentist cannot control them directly and is often forced to acknowledge patients’ emotional needs in some manner in order to provide an effective, high quality technical service. The alternative might be to refuse to treat anxious patients. Fortunately, many dentists do make conscious efforts to make themselves emotionally accessible and take time to reassure or advise patients who need it in a patient-centred or person-centred approach [41]. However, there are also many who do not instil the trust of their patients [2].

## 3. Results

Using the criterion-based concept analyses of Hupcey et al. [6] in relation to the discussion of historical precedence and the literature on dentist trust, it was apparent that the core value for patients’ trust in dentists was having their expectations met [2,3,14]. Patients, especially anxious patients, monitor behaviours and evaluate congruency between their expectations of care and actual behaviours of dentists. Trust has been shown to be dynamic (i.e., powerful and adaptive) and permitted dentists with multiple chances to meet patient expectations, depending on the importance or intensity of values behind those expectations, apropos alleviation or prevention of pain and fear [2,3,14]. Emotional risk is the main stake in trusting, as patients cannot easily evaluate the dentist’s technical work [2]. These also comprise trust in the clinical aspects of dentist–patient relationships, but at a macro level, economics also affects trust—that is, economic status and the price of treatment [14]. Trusting the dentist requires testing a relationship over time and is mostly unilateral in nature, as described in the historical background. The outcomes of loss of trust in dentistry seem more obvious than in the other four professions, since suffering is immediate in the intimacy of someone working in one’s oral cavity. Induced pain, fear and oft-resultant avoidance of care can be the outcome of distrust in dentist behaviours. However, patients do have a lot to gain from building trust with a dentist, since dentists can provide them aesthetic outcomes and help prevent pain or other suffering, if trusted (See Table 1).

Table 1 above provides a checklist of Hupcey et al.’s conceptualizations [6] as well as an evaluation of the dentistry based on dental literature and history compared and contrasted with the other professions, as tabled from their study. Although the checklist for the four other professions is twenty years old, they have not since been revised [15] and, thus, are tabled here only for conceptual comparative value with dentistry. Overall, this analysis of trust concepts in dentistry appears to support Hupcey et al.’s interdisciplinary definition of trusting relationships. However, dentistry has several unique characteristics within this definitional framework.

## 4. Discussion

### 4.1. Well-Defined Strategies to Improve Trust and Decrease Dental Anxiety

So what are responsive managerial skills of the dentist and what kind of relationship is required in lieu of the leaps of faith by persons who are very anxious about treatment?

Jacquot [42] concluded that in order to improve the dynamics of the patient–dentist relationship, consultations should be based upon mutual trust and communication. Both parties must accommodate each other’s needs and demands for equal roles in the entire process. Jacquot [42] and Moore [2] also surmised that dentists should be the ones to relinquish more control and take an active role in understanding and valuing their patients as autonomous individuals. That is to say that in working with pain and anxiety as a frequent occurrence in the dental surgery, dentists need to adjust to patient needs for patient-centred treatment and the communication skills that may be included [2,14]. This requires more focus on the patient side of the relationship instead of the dominant dentist role. Walji et al. [41] differentiated person-centred from patient-centred treatment. They argued, “Patient-centred care focuses on the disease rather than on the person, while person-centred care means an approach that focuses on the elements of care, support, and treatment that matter most to the patient Thus, person-centred care is a way of thinking and doing things that sees the people using health and social services as equal partners in planning, developing, and monitoring care to make sure it meets their needs. it requires working alongside professionals to get the best outcome.” [41].

The following topics revolve around the promotion of a more person-centred perspective in dental practice, especially in building trusting and supportive relationships with fearful patients.

#### 4.1.1. Increasing Patient Perceptions of Control in the Dental Chair

Providing fearful patients more control in their dental treatment situation is important for decreasing pain perceptions and promoting patient self-efficacy beliefs [12,32,33,43,44]. Other methods of allowing patients to feel less passive, more in control and less helpless are through the use of hand signals for rest breaks and strict adherence to patient–dentist contracts about how long the procedures will take, the order of the work by difficulty and numbers and durations of pauses [1,34,35,45,46,47,48]. Therefore, anxious patients may often need help in becoming empowered with a belief in their own ability to relate with the dentist and to feel as if they can negotiate or become more actively involved in decision-making or in the conduct of treatments. Given evidence above that dentists often fill a dominant role in the dentist–patient relationship, it would be advantageous to bring some feelings of control and influence back to the patient [49]. This includes both one-on-one patient-to-dentist contacts, as well as in the broader sense of their social roles and patient activation. Immediately, this could appear to be threatening to the dental profession, since it appears to politicise or polarise dentists and patients. Certainly, it is absurd to think that patients in large numbers would become so radical that they would start treating their own teeth. However, even at this extreme, in rare instances it has been observed that persons with dental phobia who eventually submit to therapy at dental anxiety specialist clinics after years of treatment avoidance have attempted to treat themselves, be it self-extractions or aesthetic dentistry with wax or paper fillings [1,50,51,52].

On an individual level, the concept of empowerment of passive patients can be conducted within a therapeutic process that teaches them social skills for improved self-confidence [53,54,55]. Patients who take a more active role are better at monitoring their own health and know more about dental treatment. This also saves the dentist chair time. When patients want to take more responsibility for their own dental health, the dentist is relieved of a burdening responsibility and subsequent occupational stress. Study results on this topic indicate that there is some hope that dentists could become more satisfied with their occupation [2,56]. If dentists would want to learn new communication skills that would facilitate effective mutual cooperation in the dental chair, they would be rewarded as a group by the individual patient taking more responsibility for oral health care—i.e., better patients [57]. Even if it may not be immediately apparent from some dentists’ perspectives that it is advantageous fort patients to become more active in the dental chair, in the long run, it will create another less stress and a more satisfying practice style [58,59,60]. It is important that dentists not only see it as advantageous and nonthreatening but also that they can fairly easily learn the social skills necessary for this person-centred approach [49,56,61].

The type of empowerment described above is mostly related to an interpersonal or a “micro” level where patients seek treatment based on their own premises. This assumes that dentists want to help their patients become actively involved in treatment. What if dentists find patient activation threatening and resist person-centred approaches? Empowerment at the “macro” level is a social/political process endorsing and enforcing patient-centred medicine as a moral and ethical human right. It espouses that professional dominance must give way to patient rights and informed consent as the primary force in health care systems [62,63,64,65]. What do we know from the health care literature about this type of empowerment?

One way to think of empowerment is that it is the ability of the parties (here, dentists and patients) to self-regulate their expectations of each other on the basis of increasing each “partner’s” specific self-confidence [66,67,68,69,70]. This is an understanding that lies further from the concept’s political connotation and may help dentists to understand the usefulness of adopting new attitudes. Anderson [69,71] further developed this theme and concluded that it is probably less difficult to attain empowerment among individuals with mental, physical and social resources, who are able to take responsibility for the process. Nevertheless, it is perhaps difficult to think that empowerment strategies will work for persons who lack resources necessary for coping with health problems, such as is the case for many anxious dental treatment avoiders. It seems possible that existing power imbalances in dentist–patient relationships in all too many dental practices contribute to increased marginalization of dentally phobic individuals within the health care system. This is similar to marginalization of economically poor patients [65]. It is important to reiterate, as described above, that there is documentation showing that the majority of dental treatment phobias are associated with traumatic experiences due to unfortunate or negative dentist behaviour, especially in childhood [1,51]. Too often, this important association is not emphasised. Persons with dental phobia, due to their patterns of treatment avoidance appear, from the outside, to be captains of their own dental demise and are blamed for the phenomenon, since most normal adult health values require compliance to regular dental check-ups and treatment [2]. A stunning example of this is that Danish hospitals with dental treatment units refuse to treat phobic dental avoiders with general anaesthesia on the grounds that treatment cannot be given to patients with “social reasons” for dental conditions.

In summary, patient empowerment and person-centred dentistry as a viable strategy related to improving the conditions for treatment of anxious patients at the “micro” level must, as its main objective, make dentists see sharing power with patients as one of their goals and that a person-centred perspective becomes the basis for their activities and methods [41,49,72,73,74,75]. New structural relations between clinicians and patients can make a balance of power in clinical activities possible. At a “macro” or societal level, there is a moral or ethical imperative to provide healthcare for all and to avail resources to that end [72,73,75,76]. These concepts are crucial for dental-phobic persons who have avoided treatment for many years due to anxiety, often associated with iatrogenic factors. Assimilating them back into the dental health care system requires special considerations not only by individual dentists but also by the profession and the society as a whole, since these are humanitarian issues. This implies some type of social activism. Dentists and the society at large have a social responsibility to create health care environments that are humane [77].

#### 4.1.2. Practicing Active Listening, Empathy and Relationship Building

Since Carl Rogers’ work [73,74] in the 1950s and 1960s, educators, researchers and clinicians generally agreed that effective helping relationships are characterised by the core conditions of active listening, empathy, positive regard and congruence with client, student or patient thinking. By just talking with patients and listening to them, the potential of emotional contact and support can start a therapeutic and/or preventative process for anxiety and pain in dentistry. In addition to information-giving behaviours and patient contract control mechanisms, studies by Corah et al. [45,47,48] and O’Shea et al. [46] showed that patients want the dentist to “have a calm manner”; be reassuring, empathetic, friendly and communicative; and to take patient complaints seriously, especially with respect to the relief of fear and pain. Drawing on clinical experiences as dentists, several authors have explored therapeutic communication techniques. As early as 1951, Ewen [78] encouraged the use of “Active Listening”, which he described as (1) permitting the patient to unload emotionally; (2) finding out the patient’s purpose in seeking help; (3) noting how much the patient leaves out, e.g., exactly what the patient expects of the new dentist; and (4) exploring patients’ recent dental history to judge current feelings about dentistry and dentists. Active listening requires that both patient and dentist make follow-up questioning (mirroring) in order be certain of mutual understanding [79,80]. The dentist paraphrases what he hears from the patient when responding to patient statements or questions. This lets the patient know that the dentist has heard what was said. The patient then has the opportunity to clarify in his own words. Active listening builds rapport and increases patient’s self-esteem. Patients know they are heard and feel that what they have to say is important [79,80]. This two-way communication is the basis for stepwise contractual agreements between dentist or therapist and the anxious patient. It provides instant patient security and a more active patient role.

Empathy: Research suggests that “feeling as the other person feels” plays a vital role in clinical relationships [45,81,82,83]. Empathy consists of moral, emotive, cognitive and behavioural components [84,85]. Nonverbal doctor behaviours such as leaning forward, talking without crossed-arms, positive head nodding, eye contact [82,83] and smiling [45,82,83] are all highly correlated with perceptions of empathy and, thus, are conducive to reducing patient anxiety or increasing patient perceptions of safety and understanding [45]. Not surprisingly, such responses have also been shown to facilitate communication with patients. To verbally convey empathy one must first acknowledge the patient’s emotional state or condition with an “I” statement [79,80], e.g., “I notice that you are a little anxious today.” and then continue with a suggestion, “Please let me know what would help you to feel more comfortable.” Of course, the dentist’s nonverbal behaviour must also support what is being said in framing openness and disclosures. Acknowledging stronger emotions such as fear or anger has shown to be most effective in neutralizing them [79,80,86]. A dentist’s choice of words, tone of delivery and non-judgmental behaviours are a concert of messages conveying understanding and reassurance [79,80].

Evidence about the importance of dentist empathy for helping anxious patient was also studied in a Swedish study. Kulich et al. [87] studied what general dentists [88] thought were ideal dentist traits and compared them with specific traits of anxiety specialist dentists [89]. The specialists were trained in person-centred approaches [90] to interpersonal skills and communication values [87]. The main findings were that dental phobic patients exhibit distress in social interaction with dentists during treatment [91] and that dentists with positive, empathetic personal beliefs about people and patient contacts could best help them to deal with their anxiety [89,90].

Other researchers and philosophers have speculated about the power of trust and caring in healer–patient relating [92,93,94]. If a caregiver prepares patients thoroughly and helps them to define a positive outcome, then the professional health care worker has optimally supplemented the effects of medication or procedures to be undertaken. This requires finesse in communication. Roter and Hall [95] and others [61,82,96,97,98] advocated that good clinical communication should perform the following: “(1) serve the patient’s need to tell the story of his or her illness and the doctors need to hear it; (2) reflect the special expertise and insight that the patient has into his or her physical state and well-being; (3) reflect and respect the relationship between a patient’s mental state and his or her physical experience of illness or pain; (4) maximise the usefulness of the doctor’s expertise; (5) acknowledge and attend to emotional content; (6) openly reflect the principle of reciprocity, in which the fulfilment of expectations is negotiated; and (7) help participants overcome stereotyped roles and expectations so that both participants gain a sense of power and freedom to change within the encounter.” These guiding person-centred principles promote the significance of “talking” as a term used to label the process of communicating, which has as much or more to do with listening as talking.

Relationship-building: The issues of empowerment as described in Section 4.1.1 above required some form of social awareness and political activation for an individual to be allowed empowerment and to become empowered. There are also less strategic or political methods for patients and dentists to find the right track towards maximum trust and interpersonal communication. If we see dentistry as a form of cultural setting, where patients and dentists can develop a trusting relationship over time, early exposure of children to a friendly family dentist at regular non-invasive check-ups that continues over many years has the inherent advantage of helping to inhibit fear or distrust of dentists. In the healthcare communication literature, such implicit relationship building refers to communicative acts within a trusting relationship in which the doctor or dentist implicitly encourages patients to talk about themselves, express opinions and feelings, help them understand contexts and participate in mutual decision making [95,99]. Of course, relationship building also occurs when the clinician explicitly encourages patient requests that serve to empower the patient, if a patient perceives a power imbalance. Supportive talk is an active strategy that could also be used by dentists, which includes statements of reassurance, support, empathy and verbal expressions of interpersonal sensitivity with the patient [95,99,100]. Current behavioural science coursework in dental education usually includes active listening [61,101,102]. Empathetic, warm, genuine and respectful questioning is always helpful in dental health care situations. For example, when a patient would say, “I hate going to the dentist.”, there are different ways to respond; some more helpful than others. Instead of providing a presumptive question that can be answered with “yes” or “no”, the dentist can invite the patient to be more explicit by using an open-ended question such as, “Can you tell me about past experiences or other reasons that would lead you to say that you hate going to the dentist?”; i.e., answering with questions is often more positive, focused and therapeutically efficient [80].

These verbalisations and related nonverbal behaviours facilitate patient participation because they encourage and legitimise expressions of patients’ views, needs and concerns. Thus, if asked by a dentist, a patient is more likely to state his or her preferences for treatment, since the dentist has provided an opportunity to discuss these issues. They may even feel obliged to share their views, in light of the dentist’s request. Interactions at the very beginning of medical or dental visits have been shown to significantly influence the rest of the interactional pattern of the séance [103]. Manning and Ray [103] described three forms of speech that indicate active patient participation: when patients are (1) asking questions, (2) expressing concerns and (3) providing assertive responses. They described these types of responses as having “the potential to influence the course of the interaction, elicit services from providers (e.g., information, person-centred care) and contribute to quality of treatment outcomes.” Asking questions is a straightforward process. Otherwise, an expression of concern may occur both vocally by a change in tone of voice or verbally by words such as concern, worry, afraid, frustrated, nervous or mad [103]. A patient can also to feel that they can be assertive when necessary, when feeling comfortable enough to state an opinion about health, express preferences for treatment, make suggestions or recommendations, introduce new topics for discussion, or even disagree with the clinician. Although these three behaviours are more difficult to achieve in dental surgery after dental operations have commenced, conversations and verbal contracts prior to dental operations set the emotional tone for procedures and require thoughtful consideration. Doctors or dentists who use fewer controlling and directive behaviours and who promote person-centred responses are perceived by patients as more interpersonally sensitive and more actively engaged in relationship building [49,99]. Significantly more interpersonal time spent by doctors in the clinic is devoted to providing information than to relationship building and supportive talk [49,95]. However, these person-centred acts are remembered and valued by patients, since they acknowledge patient needs [65,99]. Patients pay attention when dentists show care [65,95,99]. A fully informed patient receives the best dental care while also sharing the responsibility of risks vs. benefits with the dentist [104].

## 5. Conclusions

Trust in the dentist–patient relationship still appears to be largely dentist dominated, in spite of studies lauding the need for person-centred or patient-centred care. Persistent imbalances in power within the relationship are especially distressing for persons who are anxious about treatment and distrusting of the profession as a whole. This global distrust of dominating dentists must give way to person-centred professional strategies so that dentists and patients can tackle their dental anxiety–trust challenges, both in the public’s image of the dental profession and in clinical relationships.

## 6. Future Directions

Given the apparent need for changes in communication strategies in the dental profession, there is an immediate need for the profession to procure information that helps in honestly assessing and motivating the need for change. One avenue for future research would be to find out more about the motivations of dentists who are reluctant to adopt a more patient-centred approach in relating with patients. Another related avenue of research would be to find out more about positive cost–benefit experienced by dental professionals who already practice a patient-centred approach. These avenues of research would address what incentives there could be for dentists to change over to a patient-centred approach. This would also provide guidelines for the usefulness of learning moral and ethical values in undergraduate and continuing dental education, and it can encourage the important overall goal of improving career satisfaction and the quality of life for dental professionals.

## Figures and Tables

**Table 1:** Critical inquiries of trusting concepts in four professions compared with dentistry—a checklist.

Critical Inquiry:	Medicine	Sociology	Psychology	Nursing	Dentistry
1. Trust develops: Instantaneously? Over time?	Over time	Over time	Both	Both	Over time
2. Is ‘need’ a precondition of trust?	Yes	No	Yes	Yes	Yes
3. Does trusting always place a person at risk?	Risk discussed	Calculated risk	Yes, commonly	Yes, commonly	Emotional risk often
4. Does an individual choose to trust or not trust?	Yes, both	Yes, decision required	Yes	Yes, both	Yes, both
5. Is trust inherent or does an individual learn to trust others?	No data	No data	Both	Both	Mostly learned
6. Is person trusted by virtue of a role or by personal characteristics?	Both	Both	Personal traits	Both	Both, but role image is huge
7. Is trust unilateral, bilateral, or reciprocal?	Reciprocal	Unilateral	All	All	Mostly unilateral
8. Does establishment of trust involves testing behaviours?	No data	Yes	Yes	Yes	Yes
9. Are there different kinds of trust?	Interpersonal vs. Global	Personal vs. System	Interpersonal	Intimate vs. Caring	Emotional, Technical, Economic
10. What are the outcomes of loss of trust?	Lack rather than loss	Recontract	Lonliness/low self-image	Difficult recovery	Anxiety, pain, avoidance
11. Expected outcome: Is trust a means to an end?	No data	Depends on expectation	Intrinsic vs. Extrinsic Outcomes	Means to achieve success	Yes: nice smile, no pain

## Data Availability

Not applicable.
